# Method for Evaluating Multiple Mediators: Mediating Effects of Smoking and COPD on the Association between the CHRNA5-A3 Variant and Lung Cancer Risk

**DOI:** 10.1371/journal.pone.0047705

**Published:** 2012-10-15

**Authors:** Jian Wang, Margaret R. Spitz, Christopher I. Amos, Xifeng Wu, David W. Wetter, Paul M. Cinciripini, Sanjay Shete

**Affiliations:** 1 Department of Biostatistics, The University of Texas MD Anderson Cancer Center, Houston, Texas, United States of America; 2 Department of Molecular and Cellular Biology, Dan L. Duncan Cancer Center, Baylor College of Medicine, Houston, Texas, United States of America; 3 Department of Genetics, The University of Texas MD Anderson Cancer Center, Houston, Texas, United States of America; 4 Department of Epidemiology, The University of Texas MD Anderson Cancer Center, Houston, Texas, United States of America; 5 Department of Health Disparities Research, The University of Texas MD Anderson Cancer Center, Houston, Texas, United States of America; 6 Department of Behavioral Science, The University of Texas MD Anderson Cancer Center, Houston, Texas, United States of America; Clinica Universidad de Navarra, Spain

## Abstract

A mediation model explores the direct and indirect effects between an independent variable and a dependent variable by including other variables (or mediators). Mediation analysis has recently been used to dissect the direct and indirect effects of genetic variants on complex diseases using case-control studies. However, bias could arise in the estimations of the genetic variant-mediator association because the presence or absence of the mediator in the study samples is not sampled following the principles of case-control study design. In this case, the mediation analysis using data from case-control studies might lead to biased estimates of coefficients and indirect effects. In this article, we investigated a multiple-mediation model involving a three-path mediating effect through two mediators using case-control study data. We propose an approach to correct bias in coefficients and provide accurate estimates of the specific indirect effects. Our approach can also be used when the original case-control study is frequency matched on one of the mediators. We employed bootstrapping to assess the significance of indirect effects. We conducted simulation studies to investigate the performance of the proposed approach, and showed that it provides more accurate estimates of the indirect effects as well as the percent mediated than standard regressions. We then applied this approach to study the mediating effects of both smoking and chronic obstructive pulmonary disease (COPD) on the association between the CHRNA5-A3 gene locus and lung cancer risk using data from a lung cancer case-control study. The results showed that the genetic variant influences lung cancer risk indirectly through all three different pathways. The percent of genetic association mediated was 18.3% through smoking alone, 30.2% through COPD alone, and 20.6% through the path including both smoking and COPD, and the total genetic variant-lung cancer association explained by the two mediators was 69.1%.

## Introduction

A mediation model is a statistical approach that explores the direct and indirect effects of an independent variable (i.e., initial variable) on a dependent variable (i.e., outcome variable) by including one or more mediating variables (or mediators) [1]. In some scenarios, the mediation model can infer the causal effects from the initial variable to the mediator variable and then to the outcome variable [1]. Mediation models have been widely applied in many different fields [2], such as psychology, behavioral science, genetic epidemiology, prevention research, and political communication research. Recently, there have been efforts in using mediation analysis to dissect the direct and indirect effects of genetic variants on complex diseases in genetic variant association studies [3]–[7]. Most of these studies used data from genome-wide association (GWA) studies, in which the outcome variables were selected on the basis of case-control study design. For example, our group has applied single-mediator analysis (i.e., the Baron-Kenny procedure) to identify the mediation effects of smoking and chronic obstructive pulmonary disease (COPD) on the association between the CHRNA5-A3 genetic locus and lung cancer risk using data from a case-control GWA study of lung cancer [6]. However, ignoring the case-control study design and applying standard regressions might result in biased estimations of the indirect effects. According to recent studies of secondary phenotypes, the bias could arise in the estimations of the genetic variant-mediator association because the presence or absence of the mediator (i.e., cases and controls with respect to the mediator) is not sampled following the principles of case-control study design [8]–[12]. In this case, the mediation analysis using data from case-control studies might lead to biased indirect effect estimates, either over- or under-estimated depending on the prevalence values of outcome and mediators.

Lung cancer GWA studies have consistently shown that the CHRNA5-A3 gene cluster is strongly associated with an increased risk of lung cancer. Also, multiple studies have associated SNPs spanning this region with heavy smoking, nicotine dependence, smoking cessation and COPD [13]–[19]. Thus, there is a debate about whether the genetic variants have an impact on lung cancer risk directly or exert their effect largely through the profound effect of the variants on smoking intensity [20]–[22] or COPD [23]. Further work investigating this association concluded that there are dual pathways between the genetic variant and lung cancer association, independently via a direct effect on lung carcinogenesis and through smoking behavior [6], [7], [15], [24]–[26]. More recent studies of current smokers have shown that the genetic variants on CHRNA5-A3 gene cluster have a stronger association with cotinine levels than with self-reported smoking behavior, and suggested that the effect of the genetic variants on lung cancer risk, is largely, if not exclusively, through their effect on smoking intensity [27]–[29]. However, in an accompanying editorial Spitz et al [21] concluded that the degree to which the association is mediated by smoking is yet to be determined. Prior studies focused on one mediator (e.g., smoking) at a time, and none has studied multiple mediators simultaneously in one model. However, in reality, more than one mediator could affect the association between the genetic variant and lung cancer risk. In our previous analysis [6], we found that in single-mediator analyses smoking and COPD were mediators of the association between the single-nucleotide polymorphism (SNP) rs1051730 and risk of lung cancer. However, analyzing multiple mediators in one model could have some advantages over such single-mediator analyses [30].

**Figure 1:**
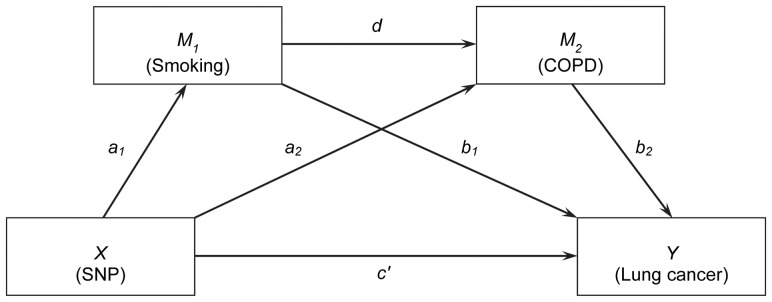
Path diagram of the multiple-mediation model for the study of SNP rs1051730, smoking behavior, COPD, and lung cancer. *X*: initial predictor variable (SNP). *M_1_*: mediator (smoking behavior). *M_2_*: mediator (COPD). *Y*: outcome variable of interest (lung cancer).

The multiple-mediation model used for the study of the SNP, smoking, COPD and lung cancer risk is depicted as a path diagram in Figure 1. The multiple-mediation model includes a three-path mediating effect through both smoking and COPD, which allows one mediator (i.e., smoking) to causally affect the other mediator (i.e., COPD) [31]. This causal association is biologically compelling because smoking is the known major risk factor for COPD [32]. The underlying assumption of this three-path mediating effect is that the individuals carrying the deleterious allele of rs1051730 are more likely to be heavy smokers, which in turn leads to a higher risk of COPD, which in turn increases the risk of lung cancer. Thus, in addition to the indirect effects passing through each of the mediators alone, we will investigate the indirect effect passing through both mediators.

To our knowledge, there has been no previous study investigating such a multiple mediation model in the case-control study design setting, in which the standard regression approach could provide biased estimations for the indirect effects as we described above. Therefore, we developed an approach to conduct a multiple-mediation analysis using the model shown in Figure 1. We conducted simulations to investigate the performance of the proposed approach, and these showed the approach can provide accurate estimates of the indirect effects. The bootstrapping approach was applied to assess the significance of the indirect effects and total effect. We also developed an approach for when the original case-control study is frequency matched on one of the mediators, as in our lung cancer case-control study where controls are frequency matched to cases with respect to smoking status. We applied the proposed approach to the multiple-mediation study of the simultaneous mediating effects of smoking and COPD on the association between SNP rs1051730 and lung cancer risk using lung cancer case-control GWA study data.

## Methods

Let *X*, *M_1_*, *M_2_*_,_ and *Y* denote the genetic variant, two mediator phenotypes, and the disease variable, respectively. We assumed binary random variables for both mediator variables and the disease variable, denoted as 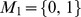, 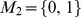, and 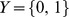, respectively, with 0 representing non-occurrence and 1 representing occurrence of the mediator phenotypes or the disease. We considered a SNP locus with two alleles: deleterious allele *A* and normal allele *a*. We first assumed a dominant or recessive genetic model for the genetic variant and also denoted it as a binary random variable, 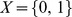. For a dominant genetic model, 0 represents genotype (*a*, *a*) and 1 represents genotypes (*A*, *a*) and (*A*, *A*); for a recessive genetic model, 0 represents genotypes (*a*, *a*) and (*A*, *a*) and 1 represents genotype (*A*, *A*). Note that if an additive genetic model was assumed, a categorical random variable 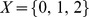 was denoted to represent genotypes (*a*, *a*), (*A*, *a*), and (*A*, *A*), respectively. Given the random variables, *X*, *M_1_*, *M_2,_* and *Y*, the association among all random variables shown in Figure 1 can be expressed using the following conditional probabilities with logistic models: 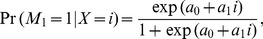
(1)

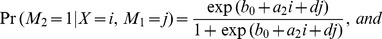
(2)

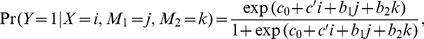
(3) where *a_0_*, *b_0_*, *c_0_*, *a_1_*, *a_2_*, *b_1_*, *b_2_*, *d*, and *c′* are regression coefficients and *i*, *j*, *k* = 0, 1. There are different indirect effects in this model [33] (see Figure 1): (i) the indirect effect passing through the mediator *M_1_*, bypassing *M_2_*, which can be assessed as *a_1_b_1_* (denoted as *IE_1_*); (ii) the indirect effect passing through the mediator *M_2_*, bypassing *M_1_*, which can be assessed as *a_2_b_2_* (denoted as *IE_2_*); and (iii) the three-path indirect effect passing through both mediators, which can be assessed as *a_1_db_2_* (denoted as *IE_3_*). Therefore, the total indirect effect passing through the mediators can be given as the summation of the above indirect effects: *a_1_b_1_*+*a_2_b_2_*+*a_1_db_2_* (denoted as *IE_t_*). The regression coefficient *c′* represents the effect of the genetic variant on the disease not mediated by either mediator and is usually called the direct effect. In general, the total effect of the genetic variant on the disease is estimated by regressing the disease variable on the genetic variant variable directly. However, the previous analysis has shown that the total effect estimated in this way could be biased when the disease variable and/or mediator variables are binary [34]. Therefore, in this study we reported the total effect (*TE*) using an alternative formula defined as the summation of the indirect and direct effects (denoted as *TE* = *IE_t_*+*c′*). In this case, the percentages of the association explained by the different mediation paths (percent mediated, *PM*) can be assessed as the specific indirect effects divided by the defined total effect, respectively, and denoted as *PM_1_* = *IE_1_*/*TE*, *PM_2_* = *IE_2_*/*TE*, *PM_3_* = *IE_3_*/*TE*, and *PM_t_* = *IE_t_*/*TE,* which represents *PM* of *M_1_* bypassing *M_2_*, *PM* of *M_2_* bypassing *M_1_*, *PM* of both *M_1_* and *M_2_*, and the total *PM* through different paths, respectively.

When the data of interest are randomly sampled from the general population, the estimations of the indirect effects and the percent mediated are accurate. However, if the data are sampled based on a case-control study design, the estimated associations among the initial variable and both mediators (i.e., *a_1_*, *a_2_*, and *d*) will be biased if standard logistic regressions are employed, which in turn, will result in biased estimations of indirect effects and the percent mediated [8]–[12]. To obtain accurate estimations of the coefficients *a_1_*, *a_2_*, and *d*, we modified the bias-correction approach proposed in our previous study [12]. Briefly, the biased coefficient estimated from the logistic regression, the prevalence values of the disease, and both mediator phenotypes can be expressed using non-linear equations. The prevalence values are obtained from the literature, and the robustness of this approach to the misspecification of prevalence values has been investigated in our previous works [12], [35]. Solving the system of non-linear equations gives us the corrected coefficients. For the purpose of the multiple-mediator model, different non-linear equations were employed to correct different coefficients. The correction approach for the regression coefficient *d* for the *M_1_*–*M_2_* association, while regressing *M_2_* on *M_1_* and *X* (see Figure 1), is given below. The correction approaches for the other parameters, namely *a_1_* and *a_2_*, are given in Text S1.

### Correction of Coefficient *d*

As stated above, the regression coefficient *d*, of the *M_1_*–*M_2_* association while regressing *M_2_* on *M_1_* and *X*, could be biased. We used the following non-linear estimating equation approach to correct the bias. Given a sample of *N* participants, of which *N_1_* are cases (*Y* = 1) and *N_0_* are controls (*Y* = 0) with respect to the disease, the odds ratio (OR) for the association between the mediators *M_1_* and *M_2_* (exp(*d*)) can be expressed as follows:
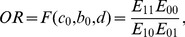
(4)where *E_kj_* is the expected number of individuals in the sample, with *M_2_* = *k* and *M_1_* = *j*, which is given as
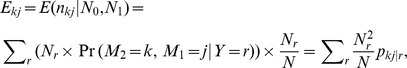
where *j*, *k*, *r* = 0, 1. The conditional probability *p_kj|r_* is written as



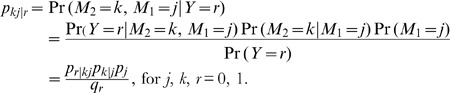



The probabilities *p_1_* and *q_1_* represent the prevalence of the mediator *M_1_* and the disease, respectively, in the general population. The conditional probabilities *p_r|kj_* and *p_k|j_* are given as functions of regression coefficients:


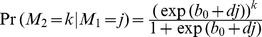
 and 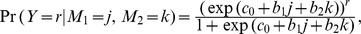
where *b_0_*, *c_0_*, and *d* are unknown coefficients of interest. Based on the conditional probabilities given above, we can write the estimated prevalences of the disease and the mediator *M_2_* as follows:
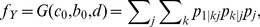
(5)

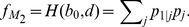
(6)


Given a sample with *N* independent individuals for a case-control study of the disease (*Y*), one can estimate the regression coefficients *b_1_* and *b_2_* as well as the biased coefficient *d* using logistic regressions based on Equations (1)∼(3). Therefore, Equations (4)∼(6) are a system of nonlinear equations with three unknown variables, *c_0_*, *b_0_*, and *d*. We employed the “fsolve” function in Matlab [36] to solve the nonlinear equation system with the use of default settings. By default, the “fsolve” function uses the trust-region dogleg algorithm, which is a variant of the Powell dogleg method [37]. The solution to this nonlinear equation system will give us the corrected estimate for coefficient *d* for the association between two mediators. As mentioned above, for brevity, the details of correction for the coefficients *a_1_* and *a_2_* were given in Text S1. We denoted the corrected coefficients as 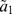, 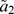, and 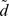. Given these corrected coefficients, the indirect effects can be estimated as *IE_1_* = 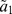 *b_1_*, *IE_2_* = 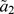 *b_2_*, and *IE_3_* = 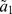 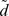 *b_2_*.

### Additive Genetic Model

When the genetic variant is assumed to be additive, special care needs to be taken. In this situation, we used a categorical random variable, 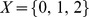, to denote the three genotypes 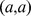, 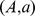, and 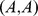. We employed the property that the biased OR obtained using logistic regression is given by the per-allele OR and adapted the approach for an additive model proposed in our previous study [35]. To obtain the true per-allele OR, we assessed biased OR in two ways. First, we obtained the biased OR_1_ by calculating the OR of SNP random variable *X* = 1 versus *X* = 0, which gives the OR for heterozygous genotype against wild-type homozygous genotype. Second, we obtained the biased OR_2_ by calculating the OR of SNP random variable *X* = 2 versus *X* = 0, which gives the OR for homozygous genotype for variant allele against wild-type homozygous genotype. On the basis of OR_1_ and OR_2_, and following the different formulas in our previous study [12], we obtained two corrected coefficients, and the final corrected coefficient for the additive genetic model is the average of these.

### Frequency-matched Case-Control Study

Frequency matching is an important and commonly used study design for known risk confounders and has been widely used in case-control studies [38]. In the analysis of real lung cancer data, because smoking is a well-known risk confounder for the association between lung cancer and other risk factors, controls were frequency matched to lung cancer cases with respect to smoking status. That is, for the multiple mediation model shown in Figure 1, the disease cases and controls are frequency matched on the mediator *M_1_*. In this scenario, frequency-matching design also contributes to bias in the estimate of the coefficients for associations among the SNP and the mediators (i.e., *a_1_*, *a_2_*, and *d*). Therefore, we adapted the approach proposed in our previous work [12] with some modifications. We first considered the calculation of 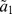. The expected numbers of individual *E_ji_* can be calculated as
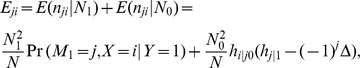
for *i = *0, 1, 2 and *j* = 0, 1.

The parameter 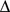 was denoted as the difference in the proportions of individuals with the presence of the mediator *M_1_* in the disease cases and controls, given as 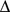 = prop(*M_1_* = 1|*Y* = 0) prop(*M_1_* = 1|*Y* = 1). In reality, the selection of controls in a frequency-matched study does not have to be perfect, that is, the proportions of individuals with the matched variables do not have to be exactly the same in the disease cases and controls (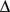 = 0). For example, in the study of lung cancer, the proportion of current smokers was 48% in lung cancer cases and 42% in controls, and the difference in the proportions was 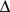 = −0.06. Therefore, the inclusion of the parameter 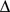 can take into account variations that occur when selecting controls that are frequency matched on the mediator, and therefore, improve the robustness of our approach. The conditional probabilities 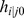 and 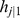 can be calculated using the same formulas given in our previous work [12]:


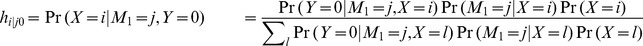
 and 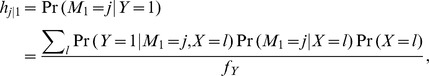
for *i = *0, 1, 2, and *j* = 0, 1.

When assessing the corrected coefficient 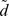, we used a similar formula to evaluate the expected numbers of individual *E_kj_*:
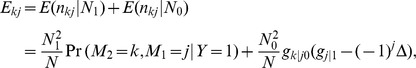
for *j*, *k* = 0, 1.

The conditional probabilities 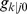 and 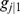 are defined as:


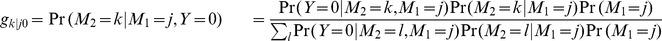
 and 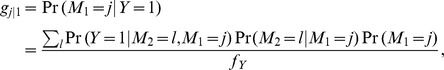
for *j*, *k* = 0, 1.

If the original disease case-control study is frequency matched on the mediator *M_1_*, the estimated value of *b_1_* will be non-significant or biased and will not represent the true association between the mediator *M_1_* and the disease. However, because the matching design considers the known risk-confounding factor at the study design phase, we typically know the associated risk. Therefore, for the frequency-matching case-control studies, we added one more constraint on the value of *b_1_*, which is fixed as the known risk coefficient (from the literature or estimated from unmatched case-control studies). Given the new formulas for *E_ji_* and *E_kj_*, one can follow the same procedure described for the unmatched study to assess the corrected coefficients 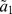 and 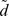, respectively. The corrected coefficient 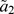
can be evaluated using the same formula of *E_ki_* that was used in the unmatched case-control study because the calculation of 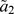
does not involve the matched mediator variable *M_1_*.

### Bootstrapping Confidence Intervals for Indirect Effects

Bootstrapping has been employed to evaluate the significance of indirect effects in a multiple-mediator model [30], [33] to overcome the difficulty in assessing standard errors for the indirect effects. In this study, we also used the empirical confidence intervals (CIs), based on a resampling-based method with replacement [39]. Given the regression coefficients *b_1_*, and *b_2_* obtained using the standard regression and the corrected coefficients 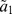, 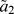, and 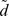 obtained using the proposed approach, the empirical CIs of the corrected individual indirect effects *IE_1_* = 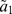 *b_1_*, *IE_2_* = 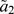 *b_2_*, and *IE_3_* = 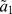 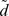 *b_2_*, as well as the total indirect effect *IE_t_* = 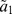 *b_1_*+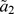 *b_2_*+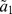 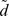 *b_2_*, were obtained by the following steps:

1. Take *B* samples with replacement from the study data, each with *n_1_* individuals from the disease cases and *n_0_* samples from the disease controls (*n = n_0_+n_1_*). Note that *n_0_*≤*N_0_* and *n_1_*≤*N_1_*, where *N_0_* and *N_1_* are numbers of cases and controls with respect to the disease in the study sample.
2. Evaluate the bootstrap regression coefficients using logistic regressions based on the bootstrap samples. Denote the bootstrap coefficients as 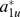, 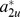, 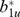, 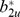, and 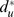, *u* = 1, 2, …, *B*. The corrected coefficients 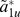, 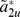, and 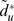, *u* = 1, 2, …, *B* are calculated by using the approaches described above.
3. The bootstrap indirect effects are assessed as 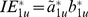, 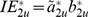, 
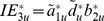
 and 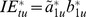
+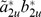
+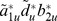, *u* = 1, 2, …, *B*. Let 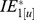, 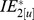, 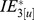 and 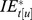 be the *u*th ordered bootstrap indirect effects estimations, respectively. Then the 100(1- 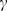)% CIs of indirect effects are given as (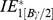,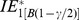), (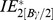,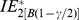), (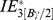,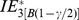), and (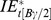,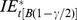), respectively.

### Simulation Approach

We performed simulation studies to investigate the performance of our approach for evaluating the indirect effects in the multiple-mediation model in a case-control study (Figure 1). To mimic the real data analysis of lung cancer, we assumed a single di-allele SNP with a minor allele frequency (MAF) of 37%. We used 14%, 24%, and 12% as the prevalence values for the disease (*Y*), the mediator *M_2_*, and the mediator *M_1_*, respectively, which approximate the prevalence values of lung cancer [40], COPD [41], and heavy smokers [42] in ever smokers. We considered two different sets of regression coefficients for the associations among the SNP, the mediators, and the disease. For the first scenario, we fixed the coefficients as *a_1_* = 0.4055, *a_2_* = 0.4055, *d* = 0.6931, *c′* = 0.4055, *b_1_* = 1.0986, and *b_2_* = 1.0986, which correspond to ORs of 1.5, 1.5, 2, 1.5, 3, and 3, respectively; for the second scenario, we fixed the coefficients as *a_1_* = 0.3365, *a_2_* = 0.3365, *d* = 0.3365, *c′* = 0.6931, *b_1_* = 0.4055, and *b_2_* = 0.4055, which correspond to ORs of 1.4, 1.4, 1.4, 2, 1.5, and 1.5, respectively. The ORs used in this simulation studies were chosen to reflect the observed ORs found in many GWA studies of common human diseases [20], [43]–[45]. According to these settings, the theoretical true values of the percentage of the total indirect effect among the association of interest are about 75% for scenario one and 32% for scenario two. For each scenario, we considered different study designs (i.e., unmatched study and frequency-matched study with respect to mediator *M_1_*) and different genetic models for the SNP (i.e., dominant, additive, and recessive genetic models). For the frequency-matched study, we also considered different values for the parameter 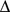 (0, ±0.05, ±0.1), which represents the difference in the proportion of individuals with the mediator *M_1_* in disease cases (*Y* = 1) and controls (*Y* = 0). On the basis of these parameters, we obtained the values for the intercept regression coefficients *a_0_*, *b_0_*, and *c_0_* for different situations.

First, we generated genotypes for a SNP using the genotype frequencies, which can be calculated from the MAF. The mediator *M_1_* values were then generated on the basis of the dataset of realizations of the SNP using Equation (1), assuming different genetic models for the SNP. Conditioned on mediator *M_1_* and the SNP values, we used Equation (2) to generate the values of the mediator *M_2_*. Last, the disease cases and controls were generated conditional on values of the SNP and both mediators *M_1_* and *M_2_* using Equation (3). In this way, we simulated a large amount of data on the population of interest and then randomly sampled 1,000 disease cases (*Y* = 1) and 1,000 disease controls (*Y* = 0). When a frequency-matched case-control study design with respect to the mediator *M_1_* was considered, the 1,000 disease cases were still sampled randomly. However, the 1,000 controls were sampled so that the proportion of the presence of the mediator *M_1_* in the controls was approximately equal to that in the cases [38]. The average results of coefficients and indirect effects reported for the simulation studies were based on 1,000 replicate datasets.

## Results

### Simulation Study

The average results of the regression coefficients *a_1_*, *a_2_*, *b_1_*, *b_2_*, *c′*, and *d* estimated using both standard logistic regression and the approach proposed in this article are reported in Table 1. In the table, the top panel shows the results for the first simulation scenario and the bottom panel shows the results for the second simulation scenario. The true regression coefficients used to generate the data are also listed in the table for the purpose of comparison. For each scenario, we investigated different study designs (unmatched and frequency-matched), different genetic models (dominant, additive, and recessive), and differences in the proportions of the matched variable (*M_1_*) between the disease cases and controls (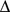 = 0, ±0.05, and ±0.1).

**Table 1:** Mean values of regression coefficients *a_1_*, *a_2_*, *d*, *c′*, *b_1_* and *b_2_* based on standard logistic regressions, as well as the corrected coefficients 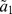, 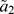 and 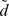 based on the proposed approach.*

**Matching**		**Genetic**	**Standard Approach**						**Our Approach**		
**Status**	Δ	Model	*a_1_*	*a_2_*	*d*	*c′*	*b_1_*	*b_2_*	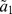	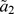	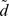
**Scenario I (true values):**			**0.4055**	**0.4055**	**0.6931**	**0.4055**	**1.0986**	**1.0986**	**0.4055**	**0.4055**	**0.6931**
**Unmatched**	**−**	**DOM**	0.4615	0.4547	0.7551	0.4041	1.0967	1.0989	0.4119	0.4069	0.6942
	**−**	**ADD**	0.4415	0.4437	0.7354	0.4095	1.1034	1.1033	0.4021	0.4037	0.6758
	**−**	**REC**	0.4353	0.4316	0.7572	0.4023	1.1031	1.1013	0.4058	0.3950	0.6964
**Matched on ** ***M_1_***	**0**	**DOM**	0.3085	0.4503	0.4408	0.4068	−0.1513	1.1010	0.3914	0.4021	0.6797
	**0**	**ADD**	0.2996	0.4409	0.4341	0.4073	−0.1906	1.0957	0.3901	0.3990	0.6786
	**0**	**REC**	0.2849	0.4397	0.4468	0.4079	−0.1389	1.1029	0.3792	0.4026	0.6865
	**−0.05**	**DOM**	0.3500	0.4502	0.5171	0.4072	0.1189	1.1003	0.3986	0.4020	0.6830
	**−0.05**	**ADD**	0.3331	0.4427	0.5046	0.4102	0.0753	1.1016	0.3976	0.4020	0.6786
	**−0.05**	**REC**	0.3329	0.4369	0.5181	0.4063	0.1326	1.1060	0.3927	0.3998	0.6850
	**0.05**	**DOM**	0.2853	0.4487	0.3852	0.4068	−0.3954	1.0989	0.3982	0.4005	0.6889
	**0.05**	**ADD**	0.2751	0.4445	0.3681	0.4030	−0.4297	1.0981	0.3982	0.4066	0.6742
	**0.05**	**REC**	0.2688	0.4389	0.3846	0.4049	−0.3824	1.0999	0.3929	0.4022	0.6889
	**−0.1**	**DOM**	0.3886	0.4554	0.5969	0.4056	0.4399	1.0999	0.3963	0.4074	0.6777
	**−0.1**	**ADD**	0.3722	0.4467	0.5809	0.4083	0.3915	1.0999	0.3998	0.4058	0.6696
	**−0.1**	**REC**	0.3719	0.4342	0.6081	0.4114	0.4570	1.1035	0.3922	0.3968	0.6902
	**0.1**	**DOM**	0.2603	0.4441	0.3165	0.4076	−0.6167	1.1080	0.4003	0.3956	0.6824
	**0.1**	**ADD**	0.2497	0.4411	0.3140	0.4094	−0.6532	1.1041	0.3987	0.3998	0.6828
	**0.1**	**REC**	0.2461	0.4401	0.3320	0.4079	−0.6077	1.1009	0.3987	0.4030	0.6960
**Scenario II (true values):**			**0.3365**	**0.3365**	**0.3365**	**0.6931**	**0.4055**	**0.4055**	**0.3365**	**0.3365**	**0.3365**
**Unmatched**	**−**	**DOM**	0.3817	0.3712	0.3530	0.6917	0.4097	0.4055	0.3432	0.3350	0.3365
	**−**	**ADD**	0.3631	0.3647	0.3473	0.6966	0.4074	0.4087	0.3384	0.3383	0.3327
	**−**	**REC**	0.3577	0.3518	0.3560	0.7030	0.4107	0.4046	0.3319	0.3256	0.3396
**Matched on ** ***M_1_***	**0**	**DOM**	0.2901	0.3711	0.3078	0.6960	−0.0720	0.4098	0.3324	0.3341	0.3345
	**0**	**ADD**	0.2733	0.3681	0.2985	0.6938	−0.1272	0.4138	0.3283	0.3414	0.3256
	**0**	**REC**	0.2730	0.3627	0.3093	0.6934	−0.0568	0.4045	0.3243	0.3367	0.3355
	**−0.05**	**DOM**	0.3601	0.3756	0.3421	0.6921	0.3405	0.4063	0.3282	0.3393	0.3264
	**−0.05**	**ADD**	0.3369	0.3665	0.3395	0.6927	0.2725	0.4130	0.3308	0.3389	0.3276
	**−0.05**	**REC**	0.3498	0.3594	0.3440	0.6990	0.3613	0.4090	0.3288	0.3330	0.3281
	**0.05**	**DOM**	0.2363	0.3734	0.2688	0.6974	−0.3943	0.4090	0.3354	0.3364	0.3286
	**0.05**	**ADD**	0.2230	0.3623	0.2677	0.6928	−0.4428	0.4087	0.3228	0.3369	0.3269
	**0.05**	**REC**	0.2215	0.3655	0.2793	0.6944	−0.3818	0.4053	0.3308	0.3395	0.3387
	**−0.1**	**DOM**	0.4759	0.3664	0.3962	0.6986	0.9516	0.4044	0.3385	0.3298	0.3238
	**−0.1**	**ADD**	0.4299	0.3636	0.3845	0.6934	0.8454	0.4015	0.3352	0.3360	0.3153
	**−0.1**	**REC**	0.4452	0.3705	0.3952	0.6961	0.9827	0.4059	0.3280	0.3445	0.3225
	**0.1**	**DOM**	0.1888	0.3710	0.2477	0.6952	−0.6665	0.4027	0.3335	0.3347	0.3337
	**0.1**	**ADD**	0.1796	0.3636	0.2461	0.6963	−0.7098	0.4049	0.3203	0.3380	0.3318
	**0.1**	**REC**	0.1750	0.3497	0.2501	0.6975	−0.6562	0.4056	0.3330	0.3235	0.3369

DOM: dominant genetic model; ADD: additive genetic model; REC: recessive genetic model

* All the results are based on 1,000 replicates, each with 1,000 disease cases and 1,000 disease controls.

For the unmatched case-control study design, when the standard logistic regressions were applied, the estimates of *c′*, *b_1_*, and *b_2_* were close to the corresponding true values, which was expected because selection of the disease cases and controls does not introduce bias in these estimations. For example, for scenario one using the dominant genetic model (unmatched study), the estimated values for *c′*, *b_1_*, and *b_2_* were 0.4041, 1.0967, and 1.0989, respectively, which were very close to the true values of 0.4055, 1.0986, and 1.0986 used for the simulations. However, the estimated values for *a_1_*, *a_2_*, and *d* were 0.4615, 0.4547 and 0.7551, respectively, which were biased compared to the true values of 0.4055, 0.4055, and 0.6931. On the other hand, the proposed approach led to estimates of 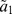, 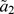, and 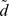 as 0.4119, 0.4069, and 0.6942, respectively, which agreed well with the true values.

When the case-control study was frequency-matched with mediator *M_1_*, in addition to the coefficients *a_1_*, *a_2_*, and *d*, the coefficient *b_1_* was also highly biased, as expected when the standard regression approach is applied; the coefficients *c′* and *b_2_* were still correctly estimated, as in the unmatched study. For example, in scenario one for frequency-matched design, when the proportion of individuals with presence of *M_1_* was higher in cases than in controls by 5% (Δ = −0.05) and the dominant genetic model was assumed, the estimated values of *c′* and *b_2_* were 0.4072 and 1.1003, respectively, which were close to the true values of simulation; however, the estimated values of *a_1_*, *a_2_*, *d*, and *b_1_* were 0.3500, 0.4502, 0.5171, and 0.1189, respectively, which were all highly biased compared to the true values. When we applied the proposed correction approach, however, accurate estimates of 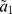, 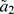, and 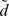 were obtained (0.3986, 0.4020, and 0.6930, respectively).

Table 2 reports the average results for the indirect effects and the percent mediated through two mediators on the effect of the genetic variant on the disease, assessed on the basis of the regression coefficient results reported in Table 1. The true indirect effects, total effect, and percent mediated are listed in the table for each scenario. We considered several specific indirect effects involved in the multiple-mediation model (Figure 1), including the indirect effect through the mediator *M_1_*, bypassing mediator *M_2_* (*IE_1_*), the indirect effect through the mediator *M_2_*, bypassing mediator *M_1_* (*IE_2_*), the three-path indirect effect through both mediators (*IE_3_*), and the total indirect effect, which is the summation of all the specific indirect effects (*IE_t_*). We also reported the total effect of the genetic variant on the disease (*TE*), as well as the percentages of the SNP-disease association explained by different paths (*PM_1_*, *PM_2_*, *PM_3_*, and *PM_t_*). For scenario one, on the basis of the coefficients used for the simulations, the true values of the specific indirect effects and the total effect were given as *IE_1_* = *a_1_b_1_* = 0.4055×1.0986 = 0.45, *IE_2_* = *a_2_b_2_* = 0.4055×1.0986 = 0.45, *IE_3_* = *a_1_db_2_* = 0.4055×0.6931×1.0986 = 0.31, *IE_t_* = *IE_1_+IE_2_+IE_3_* = 1.20, and *TE* = *IE_t_*+*c′* = 1.61, respectively; and the true values of the percent mediated by different indirect effects were given as *PM_1_* = *IE_1_*/*TE* = 0.45/1.61 = 28%, *PM_2_* = *IE_2_*/*TE* = 0.45/1.61 = 28%, *PM_3_* = *IE_3_*/*TE* = 0.31/1.61 = 19%, and *PM_t_* = *IE_t_*/*TE* = 1.20/1.61 = 75%, respectively. For scenario two, the true values of the indirect effects, total effect, and corresponding percentages mediated were assessed using the same formulas and were given as follows: *IE_1_* = 0.14, *IE_2_* = 0.14, *IE_3_* = 0.05, *IE_t_* = 0.32, and *TE* = 1.01 for indirect effects and total effect and *PM_1_* = 13%, *PM_2_* = 13%, *PM_3_* = 5%, and *PM_t_* = 32% for the percentages mediated through different indirect effects. Similar to Table 1, the top panel of Table 2 reports the results for scenario one and the bottom panel reports the results for scenario two. The results from both the standard logistic regressions and the proposed approach are reported.

**Table 2:** Mean values of different indirect effects, the total effect, and the percentages mediated for both standard regression and our approach.*

**Matching**		**Genetic**	**Standard Approach**									**Our Approach**								
**Status**	Δ	**Model**	*IE_1_*	*IE_2_*	*IE_3_*	*IE_t_*	*TE*	*PM_1_*	*PM_2_*	*PM_3_*	*PM_t_*	*IE_1_*	*IE_2_*	*IE_3_*	*IE_t_*	*TE*	*PM_1_*	*PM_2_*	*PM_3_*	*PM_t_*
**Scenario I (true values):**			**0.45**	**0.45**	**0.31**	**1.20**	**1.61**	**28%**	**28%**	**19%**	**75%**	**0.45**	**0.45**	**0.31**	**1.20**	**1.61**	**28%**	**28%**	**19%**	**75%**
**Unmatched**	**−**	**DOM**	0.51	0.50	0.38	1.39	1.79	28%	28%	21%	77%	0.45	0.45	0.31	1.21	1.62	28%	28%	19%	75%
	**−**	**ADD**	0.49	0.49	0.36	1.33	1.74	28%	28%	20%	77%	0.44	0.45	0.30	1.19	1.60	28%	28%	19%	74%
	**−**	**REC**	0.48	0.48	0.36	1.32	1.72	28%	28%	21%	77%	0.45	0.44	0.31	1.19	1.60	28%	27%	19%	75%
**Matched on ** ***M_1_***	**0**	**DOM**	−0.05	0.50	0.15	0.60	1.00	−5%	49%	15%	59%	0.43	0.44	0.29	1.17	1.57	27%	28%	19%	74%
	**0**	**ADD**	−0.06	0.48	0.14	0.57	0.97	−6%	50%	15%	58%	0.43	0.44	0.29	1.16	1.56	27%	28%	19%	74%
	**0**	**REC**	−0.04	0.48	0.14	0.58	0.99	−4%	49%	14%	59%	0.42	0.44	0.29	1.15	1.56	27%	28%	19%	74%
	**−0.05**	**DOM**	0.04	0.50	0.20	0.73	1.14	4%	43%	17%	64%	0.44	0.44	0.30	1.18	1.59	28%	28%	19%	74%
	**−0.05**	**ADD**	0.02	0.49	0.19	0.70	1.11	2%	44%	17%	63%	0.44	0.44	0.30	1.18	1.59	28%	28%	19%	74%
	**−0.05**	**REC**	0.04	0.48	0.19	0.72	1.12	4%	43%	17%	64%	0.43	0.44	0.30	1.17	1.58	27%	28%	19%	74%
	**0.05**	**DOM**	−0.11	0.49	0.12	0.50	0.91	−13%	54%	13%	55%	0.44	0.44	0.30	1.18	1.59	28%	28%	19%	74%
	**0.05**	**ADD**	−0.12	0.49	0.11	0.48	0.88	−14%	55%	13%	54%	0.44	0.45	0.30	1.18	1.58	28%	28%	19%	75%
	**0.05**	**REC**	−0.10	0.48	0.11	0.49	0.90	−12%	54%	13%	55%	0.43	0.44	0.30	1.17	1.58	27%	28%	19%	74%
	**−0.1**	**DOM**	0.17	0.50	0.25	0.93	1.33	13%	38%	19%	70%	0.44	0.45	0.30	1.18	1.58	27%	28%	19%	74%
	**−0.1**	**ADD**	0.14	0.49	0.24	0.87	1.28	11%	38%	19%	68%	0.44	0.45	0.29	1.18	1.59	28%	28%	19%	74%
	**−0.1**	**REC**	0.17	0.48	0.25	0.90	1.31	13%	37%	19%	69%	0.43	0.44	0.30	1.17	1.58	27%	28%	19%	74%
	**0.1**	**DOM**	−0.16	0.49	0.09	0.42	0.83	−19%	59%	11%	51%	0.44	0.44	0.30	1.18	1.59	28%	28%	19%	74%
	**0.1**	**ADD**	−0.16	0.49	0.09	0.41	0.82	−20%	59%	11%	50%	0.44	0.44	0.30	1.18	1.59	28%	28%	19%	74%
	**0.1**	**REC**	−0.15	0.48	0.09	0.42	0.83	−18%	58%	11%	51%	0.44	0.44	0.31	1.19	1.60	27%	28%	19%	74%
**Scenario II (true values):**			**0.14**	**0.14**	**0.05**	**0.32**	**1.01**	**13%**	**13%**	**5%**	**32%**	**0.14**	**0.14**	**0.05**	**0.32**	**1.01**	**13%**	**13%**	**5%**	**32%**
**Unmatched**	**−**	**DOM**	0.16	0.15	0.06	0.36	1.05	15%	14%	5%	34%	0.14	0.14	0.05	0.32	1.01	14%	13%	5%	32%
	**−**	**ADD**	0.15	0.15	0.05	0.35	1.05	14%	14%	5%	33%	0.14	0.14	0.05	0.32	1.02	13%	14%	5%	32%
	**−**	**REC**	0.15	0.14	0.05	0.34	1.04	14%	14%	5%	33%	0.14	0.13	0.05	0.31	1.02	13%	13%	5%	31%
**Matched on ** ***M_1_***	**0**	**DOM**	−0.02	0.15	0.04	0.17	0.86	−3%	18%	4%	19%	0.13	0.14	0.05	0.32	1.01	13%	13%	5%	31%
	**0**	**ADD**	−0.04	0.15	0.03	0.15	0.84	−4%	18%	4%	18%	0.13	0.14	0.04	0.32	1.01	13%	14%	4%	31%
	**0**	**REC**	−0.02	0.15	0.03	0.16	0.86	−2%	17%	4%	19%	0.13	0.14	0.04	0.31	1.01	13%	14%	4%	31%
	**−0.05**	**DOM**	0.12	0.15	0.05	0.32	1.01	12%	15%	5%	32%	0.13	0.14	0.04	0.31	1.01	13%	14%	4%	31%
	**−0.05**	**ADD**	0.09	0.15	0.05	0.29	0.98	9%	15%	5%	29%	0.13	0.14	0.04	0.32	1.01	13%	14%	4%	31%
	**−0.05**	**REC**	0.12	0.15	0.05	0.32	1.02	12%	14%	5%	31%	0.13	0.14	0.04	0.31	1.01	13%	13%	4%	31%
	**0.05**	**DOM**	−0.10	0.15	0.03	0.08	0.78	−12%	20%	3%	11%	0.14	0.14	0.05	0.32	1.02	13%	13%	4%	31%
	**0.05**	**ADD**	−0.10	0.15	0.02	0.07	0.76	−13%	19%	3%	9%	0.13	0.14	0.04	0.31	1.00	13%	14%	4%	31%
	**0.05**	**REC**	−0.09	0.15	0.03	0.09	0.78	−11%	19%	3%	11%	0.13	0.14	0.05	0.32	1.01	13%	14%	5%	31%
	**−0.1**	**DOM**	0.45	0.15	0.08	0.68	1.37	33%	11%	6%	49%	0.14	0.13	0.04	0.31	1.01	14%	13%	4%	31%
	**−0.1**	**ADD**	0.36	0.15	0.07	0.57	1.27	29%	12%	5%	45%	0.14	0.13	0.04	0.31	1.01	14%	13%	4%	31%
	**−0.1**	**REC**	0.44	0.15	0.07	0.66	1.35	32%	11%	5%	49%	0.13	0.14	0.04	0.31	1.01	13%	14%	4%	31%
	**0.1**	**DOM**	−0.13	0.15	0.02	0.04	0.74	−17%	20%	3%	5%	0.14	0.13	0.05	0.31	1.01	13%	13%	4%	31%
	**0.1**	**ADD**	−0.13	0.15	0.02	0.04	0.73	−18%	20%	2%	5%	0.13	0.14	0.04	0.31	1.01	13%	14%	4%	31%
	**0.1**	**REC**	−0.12	0.14	0.02	0.04	0.74	−16%	19%	2%	6%	0.14	0.13	0.05	0.31	1.01	13%	13%	5%	31%

DOM: dominant genetic model; ADD: additive genetic model; REC: recessive genetic model

* Includes the indirect effect through *M_1_* (i.e., *IE_1_* = *a_1_b_1_*), the indirect effect through *M_2_* (i.e., *IE_2_* = *a_2_b_2_*), the three-path indirect effect through *M_1_* and *M_2_* (i.e., *IE_3_* = *a_1_db_2_*), the total indirect effect (i.e., *IE_t_* = *IE_1_*+*IE_2_*+*IE_3_*), the total effect (i.e., *TE* = *IE_t_*+*c′*), and the percentages of the SNP-disease association explained by different paths (i.e., *PM_1_* = *IE_1_*/*TE*, *PM_2_* = *IE_2_*/*TE*, *PM_3_* = *IE_3_*/*TE*, and *PM_t_* = *IE_t_*/*TE*). All results are based on 1,000 replicates, each with 1,000 disease cases and 1,000 disease controls.

For the unmatched case-control study, when the standard regression approach was applied, the estimates of the specific indirect effects, as well as the total effect, were biased compared to the true values. This was expected because the coefficients used to assess the indirect effects and total effect were biased. For example, for scenario one with a dominant genetic model (unmatched study), the specific indirect effects and the total effect were given as *IE_1_* = 0.51, *IE_2_* = 0.50, *IE_3_* = 0.38, *IE_t_* = 1.39, and *TE* = 1.79, respectively, which were all biased compared with the true values of 0.45, 0.45, 0.31, 1.20, and 1.61, respectively. This, in turn, caused the percentages mediated through different indirect effects to be biased as well. For the same example, the percentages mediated by different indirect effects were estimated as *PM_1_* = 28%, *PM_2_* = 28%, *PM_3_* = 21%, and *PM_t_* = 77%, respectively. Compared with the true values of 28%, 28%, 19%, and 75%, we can observe that the percentage mediated through the three-way path of both mediators was slightly biased (*PM_3_* = 21% versus 19%) and led to a biased estimate for the total percent mediated (*PM_t_* = 77% versus 75%). However, by employing the corrected coefficients 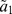, 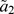, and 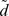
from the proposed approach to assess the indirect effects, total effect, and percent mediated, we obtained accurate estimates of *IE_1_* = 0.45, *IE_2_* = 0.45, *IE_3_* = 0.31, and *IE_t_* = 1.21 for different indirect effects, *TE* = 1.62 for the total effect, and *PM_1_* = 28%, *PM_2_* = 28%, *PM_3_* = 19%, and *PM_t_* = 75% for the percentages mediated through different indirect effects, all of which agreed well with the true values.

When the case-control study was frequency matched with mediator *M_1_*, the magnitudes of bias in the estimations of indirect effects, total effect, and percent mediated were larger than those for the unmatched study when applying the standard approach. For example, in scenario one for the frequency-matched design, when the proportion of individuals with presence of *M_1_* was higher in the cases than in the controls by 5% (*Δ* = −0.05) and the genetic model was assumed as dominant, the estimates of indirect effects and total effect were *IE_1_* = 0.04, *IE_2_* = 0.50, *IE_3_* = 0.20, *IE_t_* = 0.73, and *TE* = 1.14, respectively, which were highly biased compared with the true values of 0.45, 0.45, 0.31, 1.20, and 1.61, respectively. Accordingly, the percentages mediated through different indirect effects were estimated as 4%, 43%, 17%, and 64%, respectively, which were also biased compared with the true values of 28%, 28%, 19%, and 75%, respectively. On the other hand, the proposed approach provided estimates of *IE_1_* = 0.44, *IE_2_* = 0.44, *IE_3_* = 0.30, and *IE_t_* = 1.18 for different indirect effects, *TE* = 1.59 for the total effect, and *PM_1_* = 28%, *PM_2_* = 28%, *PM_3_* = 19%, and *PM_t_* = 74%, which were all close to the true values.

Therefore, we observed from the overall simulation results that the standard logistic regressions provided biased estimates for the coefficients *a_1_*, *a_2_*, and *d* in all situations (e.g., frequency-matched and unmatched studies) and biased estimates for the coefficient *b_1_* when the study was frequency matched on the mediator *M_1_*, which in turn led to biased estimates of the indirect effects, total effect, and percent mediated by two mediators. The magnitude of the bias in the estimations increased when the proportion difference (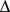) was relatively large and positive, when the original study was frequency matched on mediator *M_1_*, and when the true values used for the simulations were relatively large (i.e., scenario one versus scenario two). However, the approach proposed in this article can uniformly provide accurate estimates for the coefficients *a_1_*, *a_2_*, and *d*, and in turn provide accurate estimates for the indirect effects, total effect, and percent mediated for all situations.

### Application to the Study of Lung Cancer, COPD, Smoking and SNP rs1051730

We applied our approach to assess the mediating effects of smoking behavior and COPD simultaneously on the association between the SNP rs1051730 and lung cancer risk using a multiple-mediation model (Figure 1) based on the data from a lung cancer GWA study [6], [20], [25]. This analysis included *N_1_* = 1,153 lung cancer case subjects who were current or former smokers and *N_0_* = 1,137 control subjects frequency matched to the cases by age, sex, and smoking status. All the case and control subjects were Caucasian. Lung cancer cases were accrued at The University of Texas MD Anderson Cancer Center and were histologically confirmed. Controls were ascertained through a multi-specialty physician practice from the same area. Questionnaire data providing information on smoking were obtained by personal interview. This study was approved by the institutional review board at MD Anderson Cancer Center, and all participants provided written informed consent (LAB10-0347). We selected the number of cigarettes per day, or daily smoking quantity (SQ), as the measurement of smoking intensity. The SQ measure is categorized into two levels: SQ<25, light smokers (coded as 0); SQ≥25, heavy smokers (coded as 1) [42]. All the lung cancer cases and controls also self-reported whether a physician had ever diagnosed them with COPD, which was categorized as present or absent. The genetic variant (rs1051730) was coded as having additive effects, as in the original GWA study [20]. Since the lung cancer controls were frequency matched to the cases by smoking status, we employed the proposed approach for frequency matching with respect to the mediator *M_1_* to investigate the mediating effects of smoking and COPD. All the analyses were adjusted for age.

Table 3 reports the estimated coefficients, indirect effects, total effects, and percentages mediated for the SNP-lung cancer association obtained using both the standard and proposed approaches. As we showed in the simulation studies, the estimated coefficients *b_2_* and *c′* should be unbiased, but the estimated coefficients *a_1_*, *a_2_*, and *d* will be biased. Also, the estimated coefficient *b_1_* for smoking-lung cancer association was statistically non-significant at the 0.05 level of significance (*b_1_* = 0.1036, 95% CI = −0.0667, 0.2739) owing to the frequency matching on smoking status. To assess the corrected coefficients 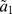, 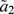, and 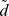, we estimated the MAF of the SNP rs1051730 from the data as 37%, and therefore, under Hardy-Weinberg proportion, the genotyping frequencies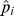, *i* = 0, 1, and 2, were calculated as 0.40, 0.46, and 0.14, respectively. The prevalences of lung cancer (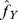), COPD (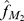), and heavy smokers (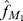) in ever smokers were obtained from the literature as 14% [40], 24% [41], and 12% [42], respectively. We further assumed the OR of association between SQ and lung cancer as 1.86, as reported by Peto et al. [46]. The 95% CIs of the coefficients for the proposed approach were obtained on the basis of our previous work [11]. To obtain the 95% CIs for different indirect effects and total effect, we performed the bootstrapping approach, as described in the Methods section, with *B* = 10,000. In addition to the estimates of the specific indirect effects, we also reported the percentage of each specific indirect effect, thus explaining the total SNP-lung cancer association.

**Table 3:** Mediation analysis results using data from a lung cancer genome-wide association study.*

	Standard Approach		Our Approach	
	Estimates	95% CIs	Estimates	95% CIs
***a_1_***	0.2477	(0.1270, 0.3684)	0.2231	(0.1024, 0.3438)
***a_2_***	0.2395	(0.0741, 0.4049)	0.2092	(0.0438, 0.3746)
***d***	0.6482	(0.4148, 0.8816)	0.6397	(0.4063, 0.8731)
***b_1_***	0.1036	(−0.0667, 0.2739)	−	−
***b_2_***	1.0919	(0.8422, 1.3416)	−	−
***c′***	0.2350	(0.1101, 0.3599)	−	−
***IE_1_*** ** (** ***PM_1_*** **)**	0.0257 (3.7%)	(−0.0181, 0.0752)	0.1385 (18.3%)	(0.0601, 0.2195)
***IE_2_*** ** (** ***PM_2_*** **)**	0.2615 (37.5%)	(0.0283, 0.4059)	0.2284 (30.2%)	(0.0398, 0.4624)
***IE_3_*** ** (** ***PM_3_*** **)**	0.1753 (25.1%)	(0.0733, 0.2995)	0.1558 (20.6%)	(0.0566, 0.3123)
***IE_t_*** ** (** ***PM_t_*** **)**	0.4625 (66.3%)	(0.1890, 0.6481)	0.5227 (69.1%)	(0.2722, 0.8476)
***TE***	0.6975	(0.3864, 0.9079)	0.7577	(0.4846, 1.0987)

CI: Confidence interval

* Both daily smoking quantity and COPD were used as the mediators in the multiple-mediator model. The 95% CIs for the indirect effects were estimated based on 10,000 bootstraps. Includes the indirect effect through smoking (i.e., *IE_1_* = *a_1_b_1_*), the indirect effect through COPD (i.e., *IE_2_* = *a_2_b_2_*), the three-path indirect effect through smoking and COPD (i.e., *IE_3_* = *a_1_db_2_*), the total indirect effect (i.e., *IE_t_* = *IE_1_*+*IE_2_*+*IE_3_*), the total effect (i.e., *TE* = *IE_t_*+*c′*), and the percentages of the SNP-lung cancer association explained by different paths (i.e., *PM_1_* = *IE_1_*/*TE*, *PM_2_* = *IE_2_*/*TE*, *PM_3_* = *IE_3_*/*TE*, and *PM_t_* = *IE_t_*/*TE*).

When the standard logistic regression approach was applied, not all three specific indirect effects were statistically significant, as evidenced by some bootstrap CIs containing zeros (Table 3). The first indirect effect carries the effect of the SNP on increasing lung cancer risk through only smoking, bypassing COPD. This indirect effect was assessed by the product of *a_1_* and *b_1_* and shown to be statistically non-significant because the 95% bootstrap CI contained zero (*IE_1_* = *a_1_b_1_* = 0.0257, 95% CI = −0.0181, 0.0752). This biased result using the standard approach was not surprising because the case-control data used for this analysis was frequency matched by smoking status (see also our simulation results for frequency-matched study design). The second indirect effect carries the effect of the SNP on increasing lung cancer risk through only COPD, bypassing smoking behavior. This indirect effect was assessed by the product of *a_2_* and *b_2_* and shown to be statistically significant, as the 95% bootstrap CI did not contain zero (*IE_2_* = *a_2_b_2_* = 0.2615, 95% CI = 0.0283, 0.4059). This result means that an individual carrying the deleterious allele for SNP rs1051730 is more likely to develop COPD, and in turn, lung cancer, independent of the individual's smoking behavior. The last indirect effect is the effect of the SNP on lung cancer risk through both smoking and COPD, which is the product of *a_1_*, *d,* and *b_2_*, and this effect was also statistically significant (*IE_3_* = *a_1_db_2_* = 0.1753, 95% CI = 0.0733, 0.2995). This result shows that the individual carrying the deleterious allele is more likely to become a heavy smoker, which in turn causes COPD and then leads to a higher lung cancer risk. The total indirect effect was evaluated as the summation of the three specific indirect effects, and as expected, it was statistically significant (*IE_t_* = *IE_1_*+*IE_2_*+*IE_3_* = 0.4625, 95% CI = 0.1890, 0.6481). Meanwhile, the direct effect of *c′* was also statistically significant (*c′* = 0.2350, 95% CI = 0.1101, 0.3599), which suggested that the SNP also affects lung cancer risk through a pathway or pathways other than smoking and COPD.

The total effect of the SNP on lung cancer risk was calculated as the sum of the direct (*c′*) and total indirect (*IE_t_*) effects (*TE* = 0.6975, 95% CI = 0.3864, 0.9079). Given the total effect, we also calculated the percentage of the SNP-lung cancer association explained by each of the specific indirect effects. The percentages mediated were estimated as 3.7%, 37.5%, and 25.1% for the three specific indirect effects, respectively, suggesting that the path through smoking alone explains 3.7% of the SNP-lung cancer association, the path through COPD alone explains about 37.5% of the association, and the path through both smoking and COPD explains about 25.1% of the association. Thus, the results obtained from the standard approach suggested that the SNP influences the lung cancer risk indirectly through two pathways (COPD only and both smoking and COPD) but not through the smoking only pathway. However, this conclusion is likely to be biased, as our simulation results showed that the case-control study design could introduce bias in the estimations of indirect effects, and furthermore, frequency matching on the basis of smoking status could conceal the true underlying association.

Therefore, we applied the new approach proposed in this article to estimate the indirect effects of smoking and COPD on the association between the SNP and lung cancer risk (see Table 3). The indirect effect of smoking, bypassing COPD, was evaluated by using the product of 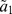 and the fixed *b_1_* value (i.e., log(1.86)) and was found to be equal to *IE_1_* = 0.1385, 95% CI = 0.0601, 0.2195, which was statistically significant. This result suggested that an individual carrying the deleterious allele is more likely to become a heavy smoker and, in turn, have a higher risk of lung cancer, independent of the individual's COPD risk. The other two indirect effects were calculated as the products of 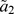 *b_2_* and 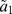 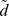 *b_2_*, respectively, and were also statistically significant (*IE_2_* = 0.2284, 95% CI = 0.0398, 0.4624; *IE_3_* = 0.1558, 95% CI = 0.0566, 0.3123). The total indirect effect (*IE_t_* = 0.5227, 95% CI = 0.2722. 0.8476) and the total effect (*TE* = 0.7577, 95% CI = 0.4846, 1.0987) were higher than those obtained from the standard approach, mainly due to the significant indirect effect through smoking only. The percentages of the SNP-lung cancer association explained by each of the specific indirect effects were also calculated and suggested that the path through smoking alone explains about 18.3% of the association, the path through COPD alone explains about 30.2% of the association, and the path through both explains about 20.6% of the association. The results obtained from the proposed approach showed that the SNP rs1051730 influences lung cancer risk indirectly through all three pathways and suggested that a higher percentage of the SNP-lung cancer association was explained by the two mediators with three pathways than indicated by the results obtained from the standard regression approach (69.1% versus 66.3%).

## Discussion

In this study, we investigated the multiple-mediation model involving a three-path mediating effect using data from a case-control study. Such multiple-mediation models have been studied previously but not in the context when the study subjects are sampled according to case-control design [31], [33]. We found that bias arises in evaluating the indirect effects if the case-control sampling study design is ignored and standard logistic regressions are applied. Therefore, we proposed an approach to correct bias in estimating coefficients from the mediation analysis and provide accurate estimates of the specific indirect effects. This approach can also be employed when the original case-control study is frequency matched on one of the mediators. We employed the bootstrapping approach to assess the significance of the indirect effects. We conducted simulation studies to investigate the performance of the proposed approach and showed that, compared with the standard approach, the proposed approach provides more accurate estimates of the indirect effects as well as of the percentages mediated by the mediators. The multiple-mediation model investigated in this study is related to directed graphic models, which have been applied to the study of genetic data. For example, Zhu and Zhang [47] investigated the association between genetic variants and multiple traits using a similar scenario as considered in Figure 1. However, their analysis was focused on testing multiple traits (e.g., primary disease and mediators) simultaneously for identifying a common genetic variant, while our study is focused on decomposing the potential direct and/or indirect effects of a genetic variant on the primary disease. Moreover, their study was based on a family-based study design, while our study is focused on a case-control study design of the primary disease in which the controls may be frequency-matched to cases with respect to one of the mediators.

We applied the approach to investigate the mediating effects of smoking and COPD on the association between the SNP rs1051730 and lung cancer risk using lung cancer case-control GWA study data where the multiple-mediation model was employed. We concluded on the basis of the results obtained from the proposed approach that the SNP rs1051730 influences lung cancer risk indirectly through all three pathways: through smoking only, bypassing COPD (18.3%); through COPD only, bypassing smoking (30.2%); and through both smoking and COPD (20.6%). The percentages mediated through different pathways (total 69.1%) obtained using the proposed approach were more correct, according to our simulation results, whereas the percentages mediated obtained using the standard approach were either under-estimated or over-estimated. Our findings that COPD mediates the effect of the SNP on the lung cancer association concurs with a previous study of the association between the SNP rs16969968 (in tight linkage disequilibrium with rs1051730) and COPD [23], in which the authors proposed that the association between the α5 subunit nAChR SNP and lung cancer could be largely explained through its relationship to COPD. Importantly, our results confirm previous findings from our group [6] that the association between the SNP rs1051730 and COPD was mediated by smoking behavior (percentage mediated = ∼40%). Thus, the study emphasizes the complex interrelationships among smoking, genes, COPD, and lung cancer.

One may argue that the use of self-reported, physician-diagnosed emphysema as a COPD measure could result in misclassification of the disease. For example, some studies have shown that when spirometry is used to assess COPD in smokers, estimates of undiagnosed COPD range from 50–80% [23], [48]–[52]. Such misclassification would lead to under-estimation of effect sizes for the association between genetic variants and COPD risk. However, a few studies suggest that the questionnaire-based approach to defining COPD is quite accurate for epidemiologic studies [53]–[55].

This study extends our previous work investigating the mediating effects of smoking and COPD on the association between the rs1051730 SNP and lung cancer using a single-mediator model [6]. However, the previous study ignored the case-control study design, which might under-estimate the indirect effect of each mediator, as well as the percent mediated by each mediator. VanderWeele et al. [7] used a weighted regression approach [56] to address the problem of case-control study design when assessing the direct and indirect effects of genetic variants on 15q25.1 on the lung cancer risk through smoking. That study focused on only a single mediator (i.e., smoking) and showed that smoking intensity only explained a small portion (∼5%) of the association between the SNP rs1051730 and lung cancer risk, which differs from the percentage we have obtained for the path through smoking only (∼18%). This difference could be due to multiple reasons. First, different types of data sets were employed in the two studies: we used only ever smokers, whereas VanderWeele et al. used both never and ever smokers for the analysis. Second, we employed a multiple-mediation model, so the indirect effect through smoking only was assessed by controlling for the other mediator, COPD, whereas VanderWeele et al. did not include COPD in their model. Moreover, the study of VanderWeele et al. used a different measure based on ORs to evaluate the percentage of the effect of the SNP mediated by smoking intensity, which assumes a rare outcome disease [56] and is not applicable to our situation because lung cancer is not rare in ever smokers. Most importantly, the difference in the results is due to the different scales used for the smoking intensity measure as the mediator variable. In the study of VanderWeele et al. [7], the square root of the number of cigarettes smoked per day was employed as a continuous mediator variable. In this case, the mediating effect can be interpreted as the effect at the square-root scale of the individual smoking one cigarette per day on the association between the SNP and lung cancer risk. In contrast, in our study we categorized the individuals into light smokers (<25 cigarettes smoked per day [mean number of cigarettes smoked per day  = 17]) and heavy smokers (≥25 cigarettes smoked per day [mean number of cigarettes smoked per day  =  38]). In this sense, the mediating effect should be interpreted as the effect of heavy smoking compared to light smoking on the association between the SNP and lung cancer risk, which as expected, would be higher than the square-root scale used in the VanderWeele study [7].

Munafo et al. [27] studied the association between genetic variants on chromosome 15q25 locus and tobacco exposure as measured by self-reported daily cigarette consumption and also based on a single measurement of cotinine levels in current smokers. They found that the genetic variants have a stronger association with cotinine level than with self-reported cigarette consumption and the per-allele increase in cotinine level indicated a per-allele increase risk of lung cancer with OR = 1.31. Since the lung cancer GWA studies suggested that the genetic variants increase lung cancer risk by 1.32 fold [20], Munafo and colleagues concluded that the association of 15q25 locus with lung cancer risk is likely to be mediated largely via tobacco exposure. Compared to our approach, this study in actuality did not perform any formal mediation analysis, but inferred the results partially based on the published data, and therefore, could not provide the percentage of the genetic variant-lung cancer association mediated by tobacco exposure. This fact was also noted by Spitz et al. [28]. The major difference in the conclusions of these two studies could also be due to the different samples (current smokers versus ever smokers) and different smoking measures (cotinine level versus smoking quantity) used.

In our study, we focused on the multiple-mediator model shown in Figure 1, which allows for the causal association of one mediator to another mediator (i.e., smoking to COPD). In our real data analysis, the causal association of smoking to COPD was known from previous studies. However, in reality, the assumed causal direction might not be known in advance and has to be obtained using theoretical justification or intuition about the area of investigation [57]. The alternative is to consider both mediators to co-vary in the model, as in a parallel multiple-mediator model [30]. Our approach can be applied to such models as well to correct the potential bias in the estimations of the indirect effects when case-control study data are employed.

The measure of percent mediated used in our study is usually applicable when the signs of the indirect and direct effects are the same [58]. However, in the multiple-mediation model, it is possible that the indirect effects, as well as the direct effect, will have different signs. In this situation, the total effect assessed by the summation of the indirect effects and the direct effect could be arbitrary, and therefore, the percent mediated by each mediator could be greater than 1 (i.e., the total effect is less than the indirect effect), negative (i.e., the total effect and the indirect effect have opposite signs), or undefined (i.e., the total effect approaches zero) [59]. One possible solution is to assess the percentages mediated using the absolute values for all indirect and direct effects [60]. Alternatively, one may use other measures, such as the measure referencing the indirect effect relative to the direct effect and the proportion of the variance in outcome variable explained by the indirect effect [61]. However, these measures might have the same issues, such as producing a negative value. In this study, we assumed there were no confounding factors mitigating associations among the SNP, smoking behavior, COPD, and lung cancer risk [7].

It should be noted that, when we refer to the direct effect, we mean the effect of the SNP on lung cancer risk directly or through pathways other than smoking and COPD.

In summary, we investigated the multiple-mediator model, which involves a three-way mediating effect from one mediator to another in a case-control study. We proposed an approach to correct the biased estimations of the indirect effects in such models due to case-control study design. The proposed approach can provide accurate estimations for indirect effects and percent mediated. It is also robust to the case-control study being frequency matched on one of the mediators. The application of the proposed multiple-mediation approach to the study of the association between SNP rs1051730 and lung cancer risk suggests that the SNP has an indirect association with lung cancer risk mainly through its effect on both smoking behavior and COPD, as well as a relatively weaker direct association with lung cancer risk. Currently, several studies are ongoing to identify genetic variants associated with smoking behaviors and COPD using existing GWA study data collected for lung cancer using simplistic regression analyses. Such studies should use more sophisticated statistical models that take into account the complex interplay of smoking, COPD, and lung cancer. Finally, additional studies that include metabolomics markers, and biochemical assays of lung carcinogens as suggested by Spitz et al. [28], and spirometry assessment among smokers as suggested by Young et al. [23], as well as together with CT scans would be needed to more accurately tease out the direct and indirect effects of the genetic variants on lung cancer risk.

## Supporting Information

Text S1 Correction of coefficients *a_1_* and *a_2_* for unmatched case-control study design.
(DOCX)
