# Supplementary material for: Method for Evaluating Multiple Mediators: Mediating Effects of Smoking and COPD on the Association between the CHRNA5-A3 Variant and Lung Cancer Risk
Source: PLoS One. 2012 Oct 15;7(10):e47705. doi: 10.1371/journal.pone.0047705 (PMC3471886; doi:10.1371/journal.pone.0047705)
Supplement: Text S1 — Correction of coefficients a1 and a2 for unmatched case-control study design. (DOCX) [file pone.0047705.s001.docx]

**Text S1. Correction of coefficients *a_1_* and *a_2_* for unmatched case-control study design**

**Correction of coefficient *a_1_***.

The regression coefficient *a_1_*, of *M_1_*-*X* association while regressing *M_1_* on *X*, could be biased. Here we provide the non-linear estimating equation approach to correct the bias.

The OR for the association between the SNP *X* and the mediator *M_1_* (exp(*a_1_*)) can be expressed as below

$OR=F(c_{0},a_{0},a_{1})=\frac{E_{11}E_{00}}{E_{10}E_{01}}$. (7)

In this scenario, *E_ji_* is the expected number of individuals in the sample with *M_1_*=*j* and *X*=*i* and is given as

$E_{ji}=E(n_{ji}|N_{0},N_{1})=\sum_{r} (N_{r}\times\Pr(M_{1}=j, X=i|Y=r))\times\frac{N_{r}}{N}=\sum_{r} \frac{N_{r}^{2}}{N}p_{ji|r}$, where *i =* 0, 1, and 2, and *j*, *r* = 0, 1. The conditional probability *p_ji|r_* is written as

$p_{ji|r}=\frac{\Pr(Y=r|M_{1}=j, X=i)\Pr(M_{1}=j|X=i)\Pr(X=i)}{\Pr(Y=r)}=\frac{p_{r|ji}p_{j|i}p_{i}}{q_{r}}$;

for *i =* 0, 1, and 2, and *j*, *r* = 0, 1.

The probabilities *p_i_*, *i* = 0, 1, and 2 represent the genotype frequencies, and *q_1_* represents the prevalence of the disease. The conditional probabilities *p_r|ji_* and *p_j|i_* are given as functions of regression coefficients:

Pr(*M_1_*=*j*|*X*=*i*) = $\frac{(\exp(a_{0}+a_{1}i))^{j}}{1+\exp(a_{0}+a_{1}i)}$ and Pr(*Y*=*r*|*X*=*i*, *M_1_*=*j*) = , (8)

where *a_0_*, *c_0_*, and *a_1_* are unknown coefficients of interest. Based on the conditional probabilities given above, we can write similar equations for the estimated prevalences of the disease and the mediator *M_1_* as follows:

$f_{Y}=G(c_{0},a_{0},a_{1})=\sum_{i} \sum_{j} p_{1|ji}p_{j|i}p_{i}$, (9)

$f_{M_{1}}=H(a_{0},a_{1})=\sum_{i} p_{1|i}p_{i}$. (10)

Note that the bias in estimating *a_1_* was not only caused by the disproportionate number of individuals with mediator *M_1_* but was also influenced by the disproportionate number of individuals with mediator *M_2_* in the primary disease case-control study. We, therefore, evaluate the regression coefficients *b_1_* and *c'* using logistic regressions in Equation (8) in order to reduce the bias due to the mediator *M_2_*. Given the biased coefficient *a_1_* estimated based on Equation (1), the solution to Equations (7), (9) and (10) will give us the corrected estimate for coefficient *a_1_* for the association between the SNP and the mediator *M_1_*, which is denoted as $\tilde{a}_{1}$. We also have tried to use an alternative approach to correcting the coefficient *a_1_* based on the estimates of *d_1_* and *a_2_*, in which the mediator *M_2_* is treated as the primary disease, as described in our previous study of secondary phenotype [[1](#_ENREF_1)]. However, we found that the alternative approach was less efficient compared with the one we have described above, based on the simulation studies. Therefore, in the current article, we only reported results based on the approach that uses the coefficients *b_1_* and *c'*.

**Correction of coefficient *a_2_***.

The regression coefficient *a_2_*, of *M_2_*-*X* association while regressing *M_2_* on *M_1_* and *X*, could also be biased. A similar non-linear estimating equation approach can be applied to correct the bias. The OR for the association between the SNP *X* and the mediator *M_2_* (exp(*a_2_*)) can be expressed as below

$OR=F(c_{0},b_{0},a{}_{2})=\frac{E_{11}E_{00}}{E_{10}E_{01}}$, (11)

where *E_ki_* is the expected number of individuals in the sample with *M_2_*=*k* and *X*=*i* and is given as

$E_{ki}=E(n_{ki}|N_{0},N_{1})=\sum_{r} (N_{r}\times\Pr(M_{2}=k, X=i|Y=r))\times\frac{N_{r}}{N}=\sum_{r} \frac{N_{r}^{2}}{N}p_{ki|r}$,

where *i =* 0, 1, and 2, and *k*, *r* = 0, 1. The conditional probability *p_ki|r_* is written as

$p_{ki|r}=\frac{\Pr(Y=r|M_{2}=k, X=i)\Pr(M_{2}=k|X=i)\Pr(X=i)}{\Pr(Y=r)}=\frac{p_{r|ki}p_{k|i}p_{i}}{q_{r}}$;

for *i =* 0, 1, and 2, and *k*, *r* = 0, 1.

The probabilities *p_i_*, *i* = 0, 1, and 2 represent the genotype frequencies, and *q_1_* represents the prevalence of the disease. The conditional probabilities *p_r|ki_* and *p_k|i_* are given as functions of regression coefficients:

Pr(*M_2_*=*k*|*X*=*i*) = $\frac{(\exp(b_{0}+a_{2}i))^{k}}{1+\exp(b_{0}+a_{2}i)}$ and Pr(*Y*=*r*|*X*=*i*, *M_2_*=*k*) = ,

where *b_0_*, *c_0_*, and *a_2_* are the unknown coefficients of interest. Based on the conditional probabilities given above, we can write similar equations for the estimated prevalences of the disease and the mediator *M_2_* as follows:

$f_{Y}=G(c_{0},b_{0},a_{2})=\sum_{i} \sum_{k} p_{1|ki}p_{k|i}p_{i}$, (12)

$f_{M_{2}}=H(b_{0},a_{2})=\sum_{i} p_{1|i}p_{i}$. (13)

Given the biased coefficients *b_2_*, *c'*, and *a_2_* estimated based on Equations (1)~(3), the solution to Equations (11), (12), and (13) will give us the corrected estimate for coefficient *a_2_* for the association between the SNP and the mediator *M_2_*, which is denoted as $\tilde{a}_{2}$.

Reference List

1. Wang J, Shete S (2011) Estimation of odds ratios of genetic variants for the secondary phenotypes associated with primary diseases. Genet Epidemiol 35: 190-200.
